# Arsenopyrite Bio-Oxidization Behavior in Bioleaching Process: Evidence From Laser Microscopy, SEM-EDS, and XPS

**DOI:** 10.3389/fmicb.2020.01773

**Published:** 2020-08-04

**Authors:** Lu Yin, Hong-ying Yang, Lin-lin Tong, Peng-cheng Ma, Qin Zhang, Miao-miao Zhao

**Affiliations:** ^1^Key Laboratory for Ecological Metallurgy of Multimetallic Mineral (Ministry of Education), Northeastern University, Shenyang, China; ^2^School of Metallurgy, Northeastern University, Shenyang, China; ^3^Zhaojin Group Co., Ltd., Zhaoyuan, China

**Keywords:** dissolution kinetics, interfacial energy, XPS, bioleaching, hydrophobicity, passive layer

## Abstract

In arsenopyrite bioleaching, the interfacial reaction between mineral and cells is one of the most important factors. The energy of the interface is influenced by the mineralogical and microbiological characteristics. In this paper, the interfacial energy was calculated, and the surface of arsenopyrite during the bioleaching process was characterized by 3D laser microscopy, scanning electron microscopy with energy-dispersive X-ray spectroscopy, and X-ray photoelectron spectroscopy, in order to assess the dissolution and oxidation behavior of arsenopyrite during bioleaching. The results showed that the contact angles of arsenopyrite were 22 ± 2° when covered with biofilms, but the reaction surface of arsenopyrite turned 103 ± 2°. However, the angle was 45–50° when covered by passive layer, which was half as that of arsenopyrite surface. The interfacial energy of arsenopyrite without biofilms increased from 45 to 62 mJ/m^2^, while it decreased to 5 ± 1 mJ/m^2^ when covered by biofilms during the leaching process. The surface was separated into fresh surface, oxidized surface, and (corrosion) pits. The interfacial energy was influenced by the fresh and oxidized surfaces. Surface roughness increased from 0.03 ± 0.01 to 5.89 ± 1.97 μm, and dissolution volume increased from 6.31 ± 0.47 × 10^4^ to 2.72 ± 0.49 × 10^6^ μm^3^. The dissolution kinetics of arsenopyrite followed the model of K_t_ = lnX, and the dissolution mechanisms were mixed controlled: surface reaction control and diffusion through sulfur layer. On the surface of arsenopyrite crystal, the oxidation steps of each element can be described as: for Fe, Fe(II)–(AsS)→Fe(III)–(AsS)→Fe(III)–OH or Fe(III)–SO; for S, As–S(-1) or Fe–S(-1)→polysulfide S→intermediate S–O→sulfate; and for As, As^–1^–S→As^0^→As^+1^–O→As^+3^–O→As^+5^–O.

## Introduction

Bioleaching is known as bio-hydrometallurgy or bio-mining and is widely applied to extract base metals and treat gold ore concentrates, where gold is associated with sulfide minerals (Schippers et al., 2013). This technique is applied to several plants in Africa, Australia, South America, and Asia (Fantauzzi et al., 2011). Arsenopyrite is the dominant arsenic-bearing sulfides in nature, which is usually covered on the gold (Ubaldini et al., 1997). The dissolution extent of arsenopyrite influences the gold recovery in the cyanidation process (Li et al., 2006). It is of great importance to understand the mechanism of arsenopyrite dissolution. The ligand of arsenopyrite is a dianion group, i.e., (AsS)^2–^, and ferrous ions are coordinated octahedrally by six anions. Each of the anions is tetrahedrally coordinated by three ferrous ions plus one other anion (Pratt et al., 1998). The surface characteristics of the mineral is critical in the bioleaching system, which controls the (bio)chemical reactions.

In bioleaching, the surfaces of arsenopyrite progress from fine cracks to pits, which progressively become wider and longer forming grooves, until the mineral is destroyed (Philippa and Carel, 2009). As a consequence of mineral bio-oxidation, sulfur, jarosites, iron oxy-hydrooxides, and scorodite are produced and cover the mineral surface (Glotch et al., 2006). The oxidized layer forms due to several possible reasons, including (Potysz et al., 2018): (i) silica gel formation, (ii) the precipitation of secondary phases, (iii) the presence of elemental sulfur or salts introduced to the solution and their deposition onto the solid, as well as (iv) the formation of intermediate compounds resulting from solid phase dissolution. All these phenomena, alone or in combination, contribute to a slowing of the dissolution process. It is therefore necessary to introduce additional adapting operations that can eliminate such factors that delay the leaching process (Potysz et al., 2018). Based on the biological and chemical characteristics, the interface between minerals and cells involves mineral surface, passive layer, and biofilm. Passive layer, densely covering the mineral surface, prevents the biochemical reactions in the bioleaching process. Many researchers focused on jarosite precipitation to increase the biochemical process (Leahy and Schwarz, 2009; Mukherjee et al., 2016). However, the mineral characteristics and dissolution kinetics under the passive layer is seldomly studied.

Surface analytical techniques such as scanning electron microscopy with energy-dispersive X-ray spectroscopy (SEM-EDS), confocal laser scanning microscopy (CLSM), X-ray photoelectron spectroscopy (XPS), and X-ray-induced Auger electron spectroscopy have been applied for this type of research. Mineral surfaces with different chemical components and roughness influence the interfacial energy. However, it is inaccurate to describe the interfacial reactions using the thermodynamic approach generally (Diao et al., 2014). In this paper, the surface of arsenopyrite was characterized in view of identifying the dissolution process and chemical compounds covering the surfaces. This study focused on bioleaching efficiency, especially on the surface characteristics during dissolution process of arsenopyrite. The dissolution of solid particles can be described as the escape of solute molecules from a solid surface and the diffusion of these molecules into the liquid phase (Hsu et al., 2009).

## Mineral and Methods

### Mineral and Microorganism Preparation

The arsenopyrite sample was provided by a mining company in Inner Mongolia, China. Chemical analyses were carried out by atomic absorption spectrometry after nitrolysis, and the results showed that the sample contained 39.4% of arsenic, 33.4% of iron, and 20.9% of sulfur. The sample was cut into cuboids with the shape of 8 × 8 × 4 mm^3^ (Supplementary Figure S1). One side of the cuboid (8 × 8 mm^2^) was first polished with silicon carbide abrasive paper (120 cw for 30 s→400 cw for 1 min→600 cw for 3 min→800 cw for 3 min→1,500 cw for 5 min→3000 cw for 5 min→5000 cw for 10 min), then polished with a cloth for 0.5 h to produce a mirror-like surface and washed with ultrapure water before placing in a bioleaching assay. The mixed culture used in this study was provided by the Bioleaching Laboratory, School of Metallurgy, Northeastern University, China. The culture was designated as HQ0211 and contains mainly *Acidiplasma*, *Acidithiobacillus*, *Leptospirillum*, and *Sulfobacillus* (Tong et al., 2007).

### Bio-Oxidization and Dissolution Experiments

The mixed culture was grown under aerobic conditions in 9 K medium with an initial pH of 1.8 at 45°C, as described by Song et al. (2015). The bioleaching experiments were performed in a 4.5 L bioleaching stirred tank reactor with 3 L mixed culture in which cell density reached ∼2.1 × 10^8^ cells ml^–1^ (statistics by blood counting chamber). The initial pH was adjusted to 1.50 ± 0.05 using 63% H_2_SO_4_. During bioleaching, the solution and cell density were controlled by replacing the mixed culture regularly. For the bio-oxidation study, the interfacial energy and the surface characteristics of arsenopyrite were studied every 2 days.

### Analysis Methods

#### Contact Angle Measurements

In this part, arsenopyrite covered with biofilms, arsenopyrite covered by passive layer, and the clean surfaces of arsenopyrite were measured. The arsenopyrite samples were taken out from the mixed culture and stored in a desiccator for 10 min. Then, the contact angles of the arsenopyrite surfaces covered by biofilms were measured using H_2_O. Later, the samples were cleaned by ultrasound for 30 min and kept in a desiccator for another 10 min in order to measure the contact angles covered by passive layer (Antoniou and Frank, 2005). Subsequently, the samples were washed with 1 M HCl and stored in a desiccator for another 10 min to analyze the contact angle of the reaction surface of arsenopyrite, as described by Yin et al. (2020). All the experiments were conducted in triplicate under the same conditions, and two to three areas on each sample were selected for measuring the contact angle.

#### Measurement of Surface Dissolution

After incubation in fresh media for several days aerobically, the samples were taken out every 2 days for analysis. Interfacial physical characterization was measured by laser microscope (LM) and OLYMPUS Stream software for 3D measurement (OLYMPUS OLS4100). The surface roughness, dissolution volume, distance, and depth of pits were analyzed by the software. In this part, 10–15 areas were measured, and the statistical data were used to reflect the surface physical characterization. A scanning electron microscope equipped with an energy-dispersive detector (SEM-EDS, SHIMADZU SSX-550) was used to examine the surface composition of the coupons.

#### Measurement of the Chemical Composition of Surface Films

The electronic structure was probed by X-ray photoemission spectroscopy (XPS) technique to analyze core levels distributions as well as valence bands (Sénémaud, 1996). It is determined by using appropriate calibration with respect to the C 1s level that is taken equal to 285.0 eV (Rocque et al., 2002). The samples were analyzed after washing by 1 M HCl. For comparison, the surface composition of a polished sample without exposure to the mixed culture was also analyzed. The results discussed were obtained from the study of the survey, As 3d and S 2p levels in the samples of bioleaching for 2, 6, and 10 days, respectively. XPS instrument was used with an ESCALAB 250Xi (Thermo Fisher Scientific, United States). The spectrometer was fitted with a monochromatized Al K_α_ X-ray source. Surveys of full range were collected using a 100 eV pass energy and a 1 eV step size. XPS studies of Fe 2p, S 2p, and As 3d core level regions were conducted at a pass energy of 10 eV and a step size of 0.05 eV.

### Data Analysis

The contact angles were calculated by the horizontal fitting method. The interfacial energy was calculated to reflect the interfacial reactions and wetting behavior in bioleaching experiments. Young’s model (Eq. 1) provides a means to analyze interfacial tension between solid and liquid.

(1) $$\cos\theta =\frac{\gamma_{S\,G}-\gamma_{S\,L}}{\gamma_{L\,G}}$$

where θ is the contact angle of an infinite system, which can be measured by sessile drop method, and γ*_*S*_*, γ*_*L*_*, and γ*_*SL*_* are interfacial tension values for solid, liquid, and solid–liquid, respectively. The values are doubtful due to the arbitrariness of γ*_*SG*_* and γ*_*SL*_*. However, many researchers still apply this approach for calculating interfacial energy.

On the other hand, three wetting mechanisms exist: dissolutive wetting (Warren et al., 1998; Zhu et al., 2007), adsorption wetting (Saiz and Tomsia, 2006), and reaction wetting (Aksay et al., 1974). By combining Eqs 2–4, a solid–liquid interface model was developed (Ren et al., 2017), which is presented as Eq. (5). The equation was derived by Zhu Dingyi and designated as ZDY equation.

(2) $$\sigma_{S\,L}=-\sigma_{L\,G}\,\cos\theta^{\prime }$$

(3) $$\sigma_{S\,G}=-\sigma_{L\,G}\,\sin\theta^{\prime }$$

(4) $$\cos\theta =\sin\theta^{\prime }+\cos\theta^{\prime }$$

(5) $$\sigma_{S\,L}=\frac{\sigma_{L\,G}}{2}\left(\sqrt{1+\sin^{2}\theta }-\cos\theta \right),0\leq \theta \leq 180{}^{\circ }$$

where θ is the contact angle of infinite system, θ′ is the contact angle of finite system. σ*_*SL*_* is the interfacial energy between solid and liquid, σ*_*L*__*G*_* is the interfacial energy between liquid and gas, and σ*_*SG*_* is the interfacial energy between solid and gas. The interfacial energy between the sample and deionized water can be calculated after measuring the values of the interfacial energy between the measured liquid and atmosphere (σ*_*L*__*G*_*) and contact angle (θ).

## Results and Discussion

### Contact Angle and Interfacial Energy Calculation

**FIGURE 1:**
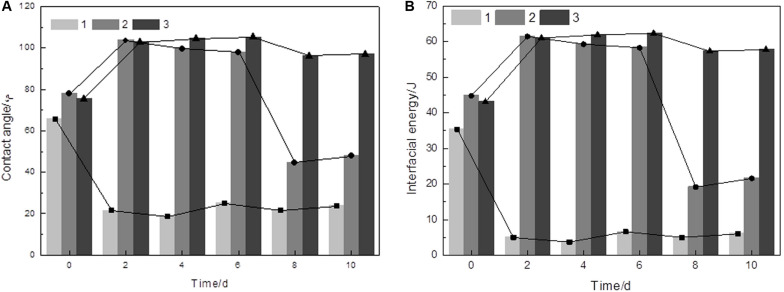
Variation of the contact angle **(A)** and interfacial energy values **(B)** of the initial arsenopyrite samples (0 day) and the arsenopyrite samples bioleached for 2, 6, 8, and 10 days. 1, arsenopyrite covered by biofilms, 2, arsenopyrite treated by ultrasonication for 30 min, 3, arsenopyrite washed with 1 M HCl. Bioleaching assays were done at an initial pH of 1.5 and with a cell density of 1.8 × 10^8^ cells/mL at 45°C.

The surface thermodynamic approach is a macroscopic and physicochemical approach that interprets the state of mineral energy. The values for the contact angle and the interfacial energy of the interfaces measured by water during bioleaching are shown in Figure 1. The values showed that the surface was hydrophilic if covered by a biofilm, but the reaction surface of arsenopyrite turned to hydrophobicity.

It was found that both contact angles and interfacial energy values for arsenopyrite were lower when covered by a biofilm than those without biofilms. The results coincide with the findings by Attia (1990) and Ohmura et al. (1993). Biofilm protects cells from environmental stress, such as desiccation, nutrient starvation, radiation, and/or oxidative stress (Flemming and Wingender, 2010). According to Zhang et al. (2018), the biofilm might indicate a role of coping with hydrostatic pressure stress and also shared the same components with leaching solution. This phenomenon explained why the contact angles were 22 ± 2° when covered by a biofilm, whereas the arsenopyrite surfaces exhibited hydrophobicity (90–105°) after acid washing. The cells may also act as a wetting agent to allow oxidation products, such as S^0^, to be dispersed in the medium, thereby allowing further oxidation (Dopson and Lindstrom, 1999). For those samples that were mainly treated by ultrasound, the contact angle initially increased from ∼77 to 100° but subsequently decreased from 98 to 45° on the eighth day. This is probably due to the presence of some oxidation by-products such as jarosite on the arsenopyrite surface (Márquez et al., 2012). The most hydrophobic surface appeared on the sixth day with the contact angle of 105° and an interfacial energy of 62 mJ/m^2^. Moreover, both contact angles and interfacial energy, for the samples after acid washing, increased at first and then decreased slightly. This might be due to that As and Fe ions were released from the arsenopyrite leaving a sulfur-rich surface (Mikhlin et al., 2006). The abiotic control groups showed that the contact angles for the samples before acid washing had a little decrease, but remained constant at ∼90° after the passive layer was removed. The interfacial energy between water and the arsenopyrite phases at each bioleaching time, calculated by applying the ZDY equation, shared the same order as contact angle.

For the arsenopyrite covered by passive layer, the contact angles and interfacial energy were attributed to the passive layer covering the surface of arsenopyrite. This indicated that the passive layer started to influence the interfacial energy after 4 days of the bioleaching process, and this effect increased significantly after 6 days. The clean arsenopyrite surface of the sixth day were the most energetic due to high electron donating characteristic (Gu et al., 2008). The electron donating characteristic can be influenced by the anisotropy of crystalline bodies in connection with their chemical compounds, surface roughness, and internal structure (Laporte, 1994). Here, we only focused on chemical compounds and surface roughness, since the contact angles were measured macroscopically. Further work should be done to measure these characteristics on the arsenopyrite surface, to analyze the arsenopyrite dissolution for bioleaching applications.

### Morphology of Arsenopyrite Surface

**FIGURE 2:**
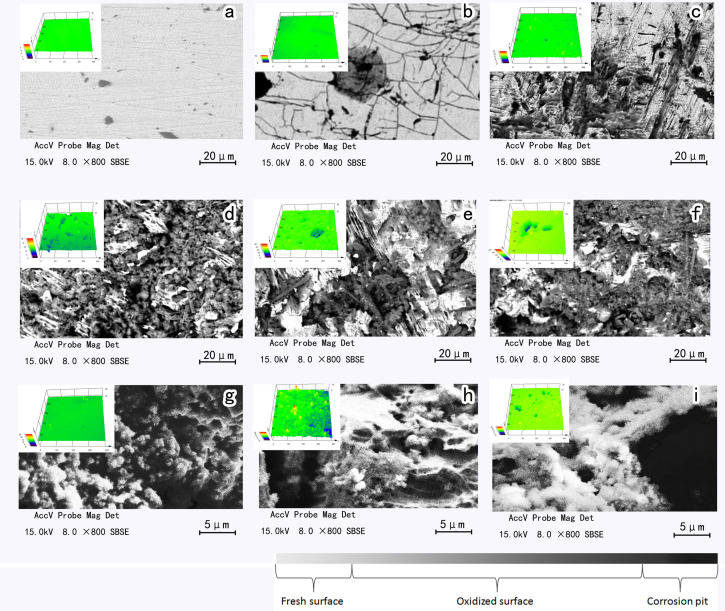
SEM and CLSM images of initial arsenopyrite **(a)**, arsenopyrite bioleached for 2 days **(b)**, 4 days **(c)**, 6 days **(d)**, 8 days **(e)** and 10 days **(f)** and the passive layer on the surface of the samples on sixth **(g)**, eighth **(h)**, and tenth **(i)** day at 45°C with initial cell density of 1.8 × 10^8^ cells/mL, pH of 1.5.

The changes in surface morphology of the arsenopyrite during bioleaching are shown in Figure 2. Arsenopyrite surfaces were appreciably influenced by the mixed culture. Based on SEM-EDS, the surface was identified and separated into fresh surface, oxidized surface, and corrosion surface. Besides, from the CLSM images (Figure 2), pits can be found on the surface after bioleaching for 6 days. For the samples without passive layer, the area of pits on arsenopyrite surface was larger than that of the passive layer covered samples (Supplementary Figure S2).

For the surfaces covered by jarosite and some oxidation products, the dissolution process still proceeded under the passive layer. In Figure 2B, it can be seen that the arsenopyrite surface was oxidized, and irregular lines and small gray points appeared on the surface. The non-homogeneous surface may be the result of low crystallinity of arsenopyrite (Márquez et al., 2012), and the overall sulfide mineral dissolution may be dominated by surface reactions with Fe^3+^ in the early stage of bioleaching (Edwards et al., 2001). On the fourth day, the streaks at the surface of the arsenopyrite became rougher than that of the second day accompanied by a few shallow pits, appearing as black in SEM images (Medved et al., 2004). The surface became increasingly rough with many wrinkle-shaped gullies appeared on it. On the eighth day, the interface became rougher, and many deep pits were detectable. The amount and area of the pits on the 10th day were larger than on the 8th day. The detailed information for arsenopyrite surfaces is shown in Table 1.

**TABLE 1:** Characteristics of arsenopyrite surfaces during bioleaching (pH 1.5, 45°C with an initial cell density of 1.8 × 10^8^ cells/ml).

**Time/d**	**Roughness/μm**	**Pit distance/μm**	**Pit depth/μm**	**V_*dissolution*_/μm^3^**
0	0.0340.009	–	–	–
2	0.0450.015	11.651.596	0.3960.109	63,0824,698
4	0.0950.032	21.953.892	3.5880.983	126,91410,594
6	0.4780.153	46.5115.983	10.7736.689	229,33318,695
8	0.8110.356	150.2535.896	61.76016.958	655,63635,894
10	5.8921.968	229.3550.978	108.00925.693	2,723,517489,351
Control	12.393.018	210.9569.183	104.23115.203	6,124,291329,079

**The samples were washed with 1 M HCl after taking out from the bioleaching assay. Control, passive layer controlled samples.**

As is shown in Table 1, all measured parameters increased exponentially during exposure. At the end of bioleaching, the surface roughness of clean arsenopyrite amounted to 5.9 ± 2.0 μm, and values for the distance and depth of pits at the end of bioleaching were ∼230 and ∼108 μm, respectively. The dissolved volume of arsenopyrite was ∼2.7 × 10^6^ μm^3^ at the end of bioleaching. The surface roughness of arsenopyrite increased from 0.03 ± 0.01 μm to 5.9 ± 2.0 μm. The most significant increase appeared on the 10th day, which was as much as six times higher than that of the 8th day. For the passive layer controlled samples, the surface roughness, pit depth, and dissolution volume were 12.39, 210.95 μm, and 6.1 × 10^6^ μm^3^, respectively. It means that passive layer had little effect on the depth of corrosion pit, while the dissolved volume was significantly affected. From the data obtained here, the passive layer affected the dissolution volume but had no significant effects on pit depth. As a result, the exposure probability of gold would be influenced by passive layer, but the corrosion depth of arsenopyrite would not be affected in bioleaching, theoretically. This shares the same opinion with Jones et al. (2003) that the overlayer did not prevent continued alteration of arsenopyrite.

The weight contents of Fe, As, and S at different areas were determined by EDS (Supplementary Table S1). From these data, it became clear that sulfur was enriched in the pits within the first 4 days of the bioleaching process, and it was also accumulated on the surface. The values of Fe/As/S ranged from 0.0185 to 0.1099. The EDS data changed remarkably during bioleaching and reflected the oxidization process roughly. For the fresh and oxidized surfaces, the maximum value appeared on the sixth day, which corresponds to the highest interfacial energy. This clearly indicates that the interfacial energy is influenced by the fresh and oxidized surfaces. It also explains why the interfacial energy increased during bioleaching. Since the ratio of Fe, As, and S can be used to describe the oxidization of the arsenopyrite (Zhang et al., 2019), the detailed chemical states of the surface during dissolution process are still necessary to be revealed.

### XPS Study of Arsenopyrite

According to the morphology results obtained, the dissolution extent of arsenopyrite increased almost 10 times from 6th to 10th day. The deepest pits of the samples on 2nd, 6th, and 10th day were analyzed to reveal the surface chemical compositions influenced by bioleaching.

The high-resolution spectra for iron, arsenic, and sulfur species were recorded. The survey (full range) XPS spectra of the arsenopyrite samples are presented in Supplementary Figure S3. The spectra were corrected by shifting all peaks to the adventitious C 1s spectral component binding energy set to 284.8 eV. The O 1s peak may be related to air contamination and/or surface oxidation during bioleaching. The peak of N might result from the remaining cell products, and its content was 1.66–2.48 wt%. The peak intensity of Fe, As, and S changed considerably during bioleaching. According to Yin et al. (2016), Fe–(AsS) is easier to be oxidized than As–S, it is necessary to analyze the valence and bonding situations of these elements to reflect the oxidation tendency. The interfacial energy can reflect the oxidation tendency, and this explained why the interfacial energy increased during bioleaching, since the Fe–(AsS) bonds are easier to be oxidized than As–S bonds (Yin et al., 2016).

#### Fe 2p Spectra

**FIGURE 3:**
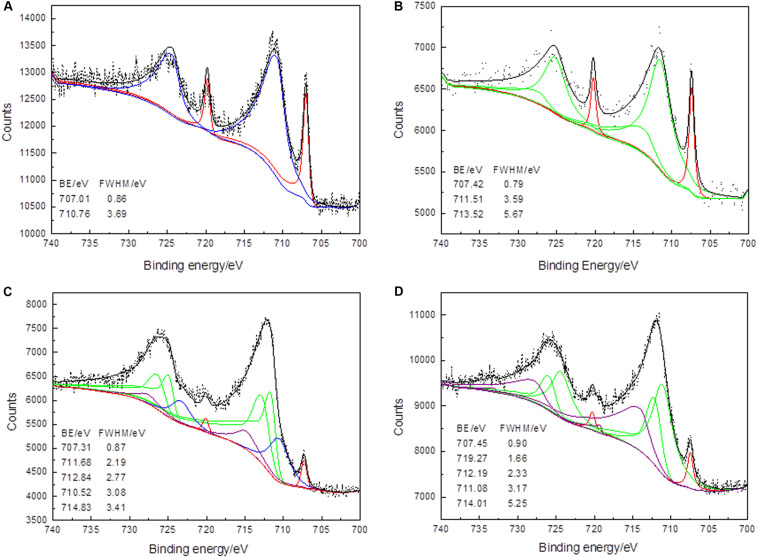
XPS Fe 2p spectra of arsenopyrite before **(A)** and after bioleaching with mixed culture for 2 days **(B)**, 6 days **(C)**, and 10 days **(D)** at 45∘C and initial pH 1.5. (Short dots – the experimental data. Fits to data – black The curves in red, blue, green, and purple present the spin-orbit split peaks of Fe(II) –(AsS), Fe(III) –(AsS), Fe(III) –OH, and Fe(III) –SO)

Fe in the dichalcogenides is in low spin state, and a single photoemission peak should result in the Fe 2p spectrum (although spin–orbit split) (Harmer and Nesbitt, 2004). The Fe 2p conventional XPS spectra of the arsenopyrite during the course of bioleaching together with the original samples were recorded and put together in Figure 3. The conventional Fe 2p spectra of the arsenopyrite surface at the beginning of the bioleaching varied from those at the middle and later period. The binding energy of Fe 2p_3__/__2_ peaks are located at ∼707.2, 710.5, 711.4, 712.4, 713.5, 714.8, and 719.2 eV, representing Fe(II)–(AsS), Fe(III)–(AsS), Fe(III)–OH, and Fe(III)–SO, respectively.

The narrow peak detected at ∼707.2 eV in the Fe 2p spectra was due to singlet Fe(II) in arsenopyrite phase (Mikhlin et al., 2006). Fe(II)–(AsS) and Fe(III)–(AsS) were the main chemical states in the sample before bioleaching, weighing 27.42 and 72.58 at%, respectively. This means that a large amount of arsenopyrite had been oxidized by the action of air. This may because the oxidation sequence is Fe > As = S in abiotic oxidation (Zhu et al., 2014; Deng et al., 2018). Nesbitt et al. (1995) characterized the surface of arsenopyrite oxidized in air; Fe(II)–(As–S) and Fe(III)–(As–S) were found on the surface. Interestingly, Fe(III)–(As–S) was detected on the surface of arsenopyrite before bioleaching, and the sample that was bioleached for 6 days. Fe(III)–OH can be detected on the surface bioleached for 2, 6, and 10 days, weighing 87, 71.5, and 61.98 at%. For the samples that were bioleached for 6 and 10 days, Fe(III)–OH contained two binding energies located at 711.68 and 712.84 eV. Fe(III)–SO was detected on the arsenopyrite surfaces after bioleaching for 6 days. At the end of the bioleaching process, Fe(III)–OH and Fe(III)–SO were the main iron-containing compounds with atomic percentages of 61.98 and 33.06%, respectively. The oxidation steps of Fe can be described as Fe(II)–(AsS)→Fe(III)–(AsS)→Fe(III)–OH, or Fe(III)–SO. This oxidation step corresponds with that of Corkhill et al. (2008) in the presence of *L. ferrooxidans*. However, the difference is that the mixed culture used in this experiment increased the bioleaching efficiency by decreasing the transfer step of Fe(II)–(AsS)→Fe(III)–(AsS).

#### S 2p Spectra

**FIGURE 4:**
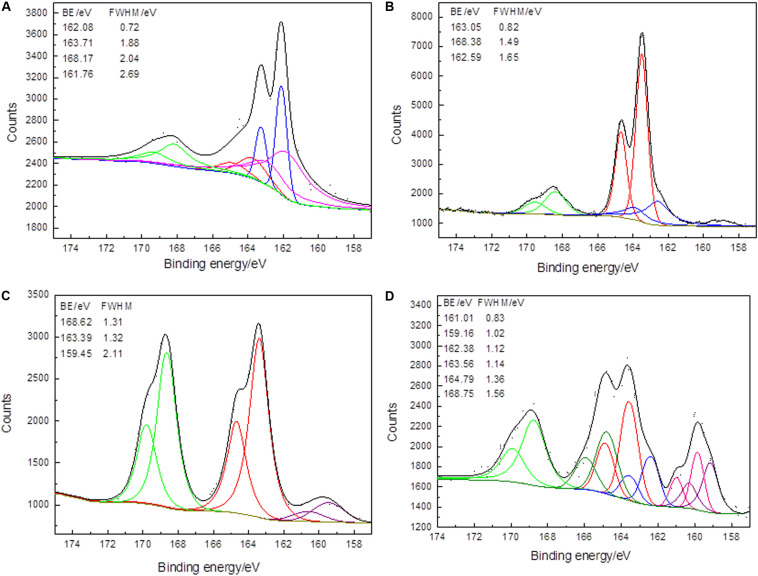
XPS S 2p spectra of arsenopyrite before **(A)** and after bioleaching with mixed culture for 2 days **(B)**, 6 days **(C)**, and 10 days **(D)** at 45∘C and initial pH 1.5. Short dots present the experiment data. Fits to data and the background are in black and dark yellow curves. The curves in red, blue, green, olive, pink, and purple present the spin-orbit split peaks of S_n_^2–^, As–S, SO_4_^2–^, intermediate S–O, mono-sulfides, and Fe–S.

Figure 4 presents the conventional S 2p spectra of the arsenopyrite surface before bioleaching and after bioleaching for 2, 6, and 10 days. They displayed strong bulk signal representing 5–29% S on the surface. S 2p signals resulted from the spin-orbit signal of S atoms from bulk dimers. The binding energy of S 2p_3__/__2_ peaks were located at ∼159.4, 162.3, 162.5, 163.5, 164.7, and 168.5 eV, representing Fe–S, As–S, S^0^, polysulfide, intermediate S–O species, and sulfate, respectively (Mikhlin et al., 2006; Zhu et al., 2014).

Before bioleaching, As(-1)–S contained 22.23 wt%, and at the end of the bioleaching, only 10.62 wt% was detected. Besides, it should be noticed that the peak position of S 2p moved to a position where binding energy increased. The peak shifts were especially significant for As–S and sulfate. For the As–S, the binding energy increased from 162.08 eV (initial sample, 22.23 wt%) to 162.43 eV (10 days, 10.62 wt%). The binding energy of the initial sample was ∼0.4 eV lower than that of the other two samples. It showed that, for the samples that were treated for 2 and 10 days, the electron density of S atoms might be slightly lower than that of the S atoms of the initial arsenopyrite. Thus, As–S bonds were greater than in the initial samples, consequently the increased binding energy. On the other hand, sulfate could be detected in all samples, with binding energies of 168.17 (13.86 wt%), 168.38 (16.17 wt%), 168.62 (40.63 wt%), and 168.76 (25.86 wt%) eV. The increase by 0.59 eV during the bioleaching implies that the S–O bonds of sulfate were more stable with the process of bioleaching. However, the chemical states of S were complex at the end of the bioleaching, which indicated that at the end of the process, the surfaces were bio-corroded by the microorganisms and the oxidization products were accumulated in the pits.

In the original arsenopyrite, Fe, As, and S atoms are combined as Fe(II)–(As–S) or Fe(II)–(S–As). Since S is more electronegative than both Fe and As, the S atom reacts with one As atom or three Fe atoms when an Fe–S or As–S bond is broken (Gupta and Sen, 1974). Polysulfide existed in the initial samples (26.58 wt%) and in the samples treated for 2 days (60.62 wt%) and 6 days (51.61 wt%). The content increase in polysulfide implied that it was produced during bioleaching. Besides, polysulfide disappeared at the end of bioleaching, meaning that at the end of bioleaching, it was oxidized to S–O (intermediate S–O or sulfate). S^0^ was only detected on the surface bioleaching for 2 days (12.16 wt%). The oxidation step of the sulfur moiety can be described as Fe–S and/or As–S→polysulfide or S^0^→intermediate S–O→sulfate. Corkhill and Vaughan (2009) pointed out that it is still unclear whether the accumulation of S^0^ will prevent further oxidation of the arsenopyrite surface. In this study, S^0^ was detected, while further oxidation was not affected, most likely due to presence of sulfur oxidizers, e.g., *Acidithiobacillus* and *Sulfobacillus* (Rohwerder et al., 2003). Besides, the existence of polysulfide S resulted in the surface hydrophobicity and increased interfacial energy, which led to increase in microbial attachment and biofilms formation.

#### As 3d Spectra of Arsenopyrite During Bioleaching

**FIGURE 5:**
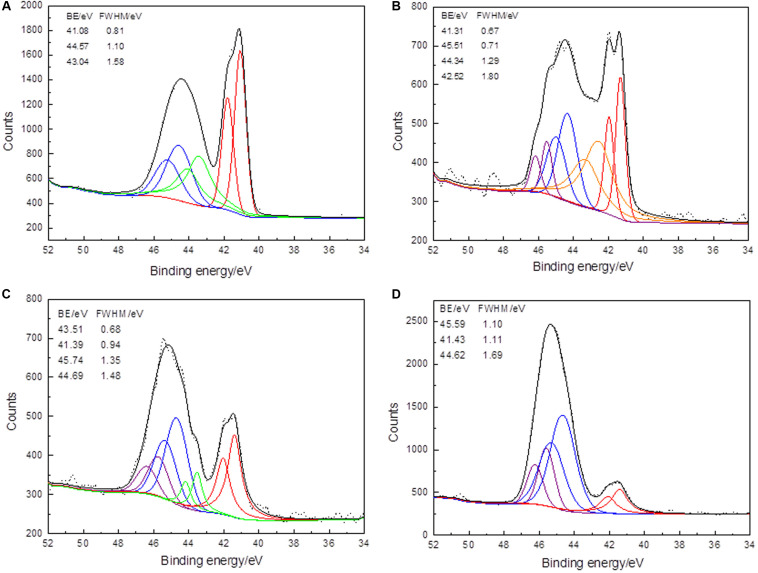
XPS As 3d spectra of arsenopyrite before **(A)** and after bioleaching for 2 days **(B)**, 6 days **(C)**, and 10 days **(D)** at 45°C with initial pH 1.5. Short dots present the experiment data. Fits to data and the background are in black and dark yellow curves. The curves in red, blue, green, olive, and purple present the spin-orbit split peaks of As–S, As(III)–S, and As(V)–O.

The As 3d conventional XPS spectra for arsenopyrite are shown in Figure 5. The signal from As atoms in the bulk phase of arsenopyrite surface was appreciably different due to bio-oxidation. The binding energies for As 3d peaks were located at ∼41.3, 42.5, 43.5, 44.5, and 45.6 eV, which represent As–S, As(0), As(I)–O, As(III)–O, and As(V)–O, respectively.

The As 3d signals of the arsenopyrite surface after bioleaching for 2 and 6 days were more complex than those of the initial and last samples. This reflects the increased in vibrational strengthening or discording. For the arsenopyrite before bioleaching, the bulk As 3d peak was 41.08 eV (As–S bond, 39.53 wt%), together with another two peaks located at 43.40 (32.16 wt%) and 44.57 eV (28.31 wt%), which were significantly higher than that excepted for As in arsenopyrite (40.7, 41.2, and 42.1 eV) (Richardson and Vaughan, 1989; Nesbitt and Muir, 1998; Fantauzzi et al., 2011). This shows that the samples were already oxidized during surface preparation, which was reflected by the detection of Fe(III)–(AsS). For the sample that was bioleached for 2 days, the main chemical states of As were As(0) (33.97 wt%) and As(III)–O (29.29 wt%), accompanied by As(V)–O (20.15 wt%). For the sample exposed for 6 days, the bulk peaks represented As(III)–O with 20.97 wt%, As(V)–O with 32.39 wt%, and As–S with 33.66 wt%, accompanied by a minor peak of As(I)–O (12.99 wt%). For the samples bioleached for 10 days, the bulk peaks were As(III)–O with 59.36 wt% and As(V)–O with 24.26 wt%, together with a minor peak of As–S with 15.98 wt%. According to the modified Auger parameter α’, the chemical state of arsenic is similar to arsenic in scorodite (FeAsO_4_.2H_2_O), a hydrated iron(III) arsenate (Wagner et al., 1979; Moretti, 1998; Fantauzzi et al., 2006).

The main differences between the samples during bioleaching are that the signals of As 3d with low binding energies, e.g., the disappearance of the As(I)–O, and the weakening of the As–S peaks. The sensitive As 3d spectra varied with the increasing binding energies, implying that the surface has been reconstructed during the bioleaching. The binding energy and the weight content of As(V)–O (45.74 eV) of the sixth day were the highest during bioleaching; it may be the result of the dissolution of arsenopyrite. It is also possible that some other intermediate steps were also involved, such as As(II) and As(IV) (Corkhill et al., 2008). However, from the XPS spectra of the arsenopyrite interface during this bioleaching, the arsenic oxidation step can be described as: As^–1^–S→As^0^→As^+1^–O→As^+3^–O→As^+5^–O.

### Dissolution Kinetics

**FIGURE 6:**
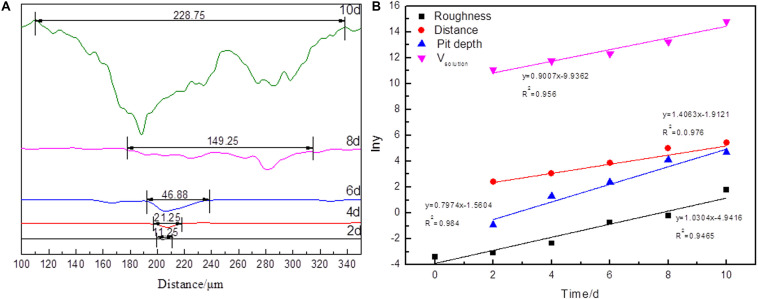
The profile of the pits **(A)** in arsenopyrite slice and plot of Kt versus time for arsenopyrite dissolution (x = time) **(B)** after bioleaching for 2, 4, 6, 8, and 10 days with mixed culture at pH 1.5, 45°C with an initial cell density of 1.8 × 10^8^ cells/mL. The arsenopyrite slices were washed with 1 M HCl measurement. The curves were measured by a laser microscope and analyzed with a OLYMPUS Stream software for 3D measurement.

The depth profiles and dissolution kinetics models of the pits during bioleaching are shown in Figure 6. There were one to six pits in every 2,500 μm^2^, but because the dissolution was inhomogeneous, the pit closest to the mean value was chosen to analyze the dissolution process. Figure 6A shows that the sample was smooth, and almost no sign of pit was detected on the second day with the distance of 11.25 μm and the depth of ∼0.3 μm. On the fourth day, a little sign of pits was detectable with the distance of 21.25 μm and depth of ∼3 μm. The surface became rougher, and the pits became deeper over bioleaching time, and the pits increased significantly after the sixth day. Since cells attached and propagated on the surfaces of the arsenopyrite (Ramírez-Aldaba et al., 2017), biofilms accumulated and the bio-oxidation ability increased. As shown in Table 1, the width of these pits increased from ∼11 to 229 μm. The depth of pits proliferated with time, ranging from ∼75 to 143 μm at the end of bioleaching process. Consequently, the bio-oxidation or dissolution reactions occurred rapidly beneath the biofilm (Parthipan et al., 2017).

The ratio of the distance to depth of the pits decreased from 29.42 to 2.12. It means that the depth increased faster than its distance. The significant increase in the values of distance and depth of pits also appeared in the samples bioleaching for 10 days. All the indices indicated that the dissolution process occurred at different rates.

Surface roughness, dissolution volume, and information of pits are the important parameters to reveal the dissolution process. In Figure 6B, the fitting function and correlation coefficients are also shown in the plot. The results indicated that all dissolution indices via bioleaching time can be described as follows:

(6) $$y={\text{e}}^{\text{a}\times t+\text{b}}$$

The dissolution kinetics follow the model in Eq. (7):

(7) K=tlnX

It should be noted that the kinetics model is similar to the model proposed by Sokiæ et al. (2009). The difference might have arisen from the difference of objects and physical quantities. In this study, the surface chemical composition of arsenopyrite was measured instead of the concentrations of ions. Sokiæ et al. (2009) proposed that the dissolution mechanism was surface reaction controlled in the initial stage, and later, it was controlled by lixiviant diffusion through a sulfur layer. However, in this study, elemental sulfur was only detected on the surface treated for 2 days, but later, the surface was covered by Fe(III)–OH and scorodite. But based on the dissolution volume and information of pits, arsenopyrite dissolution was controlled by the surface reaction.

## Conclusion

Based on the dissolution and oxidation behaviors of arsenopyrite during bioleaching, the following conclusions can be drawn:

1. In the course of bioleaching, the surface become hydrophilic with the contact angles of 22 ± 2°, if the samples were covered by biofilms. However, the reaction surface was hydrophobic, and the contact angle increased to ∼100° with the interfacial energy of ∼60 mJ/m^2^.
2. The surface roughness, pit distance, pit depth, and dissolution volume of arsenopyrite increased exponentially, and the dissolution kinetics follows K_t_ = lnX. The dissolution mechanism is surface reaction controlled.
3. The oxidation steps of Fe, S, and As in arsenopyrite bio-oxidation can be described as follows: For Fe, Fe(II)–(AsS) oxidized to Fe(III)–(AsS) before bioleaching. The Fe(III)–(AsS) cleaved and formed Fe(III)–OH and finally bond with –SO during bio-oxidation. On the other hand, S in the original samples existed as (AsS)^2–^ or bonded with Fe. In addition, polysulfide and S^0^ was formed during bio-oxidation and then oxidized to intermediate S–O, which ultimately oxidized to stable sulfate. For As, in the initial samples, it existed as (AsS)^2–^ or As^+1^–O. During bioleaching, they were oxidized to As^0^, As^+1^–O, and As^+3^–O. At the end of the bioleaching process, it existed as As^+5^–O.

## Data Availability Statement

All datasets generated for this study are included in the article/Supplementary Material.

## Author Contributions

All authors listed have made a substantial, direct and intellectual contribution to the work, and approved it for publication.

## Conflict of Interest

PM was employed by Zhaojin Group Co., Ltd.

The remaining authors declare that the research was conducted in the absence of any commercial or financial relationships that could be construed as a potential conflict of interest.
