# Supplementary material for: Arsenopyrite Bio-Oxidization Behavior in Bioleaching Process: Evidence From Laser Microscopy, SEM-EDS, and XPS
Source: Front Microbiol. 2020 Aug 4;11:1773. doi: 10.3389/fmicb.2020.01773 (PMC7417448; doi:10.3389/fmicb.2020.01773)
Supplement: Supplementary file 1 [file Data_Sheet_1.DOCX]

## Supplementary Figures


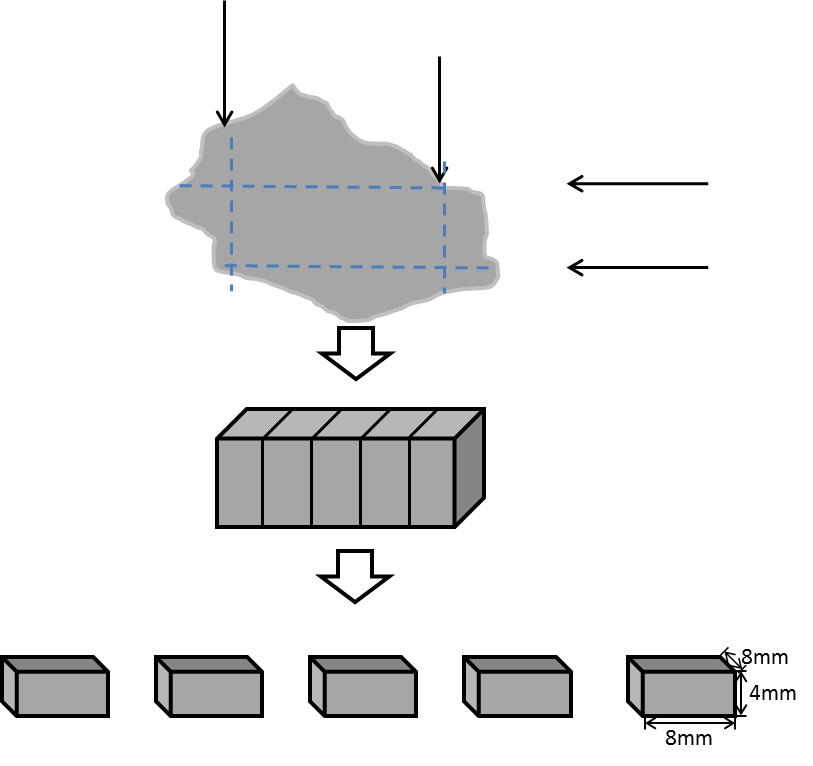


**Supplementary Figure S1.** Schematic diagram of pyrite selection and surface preparation

**
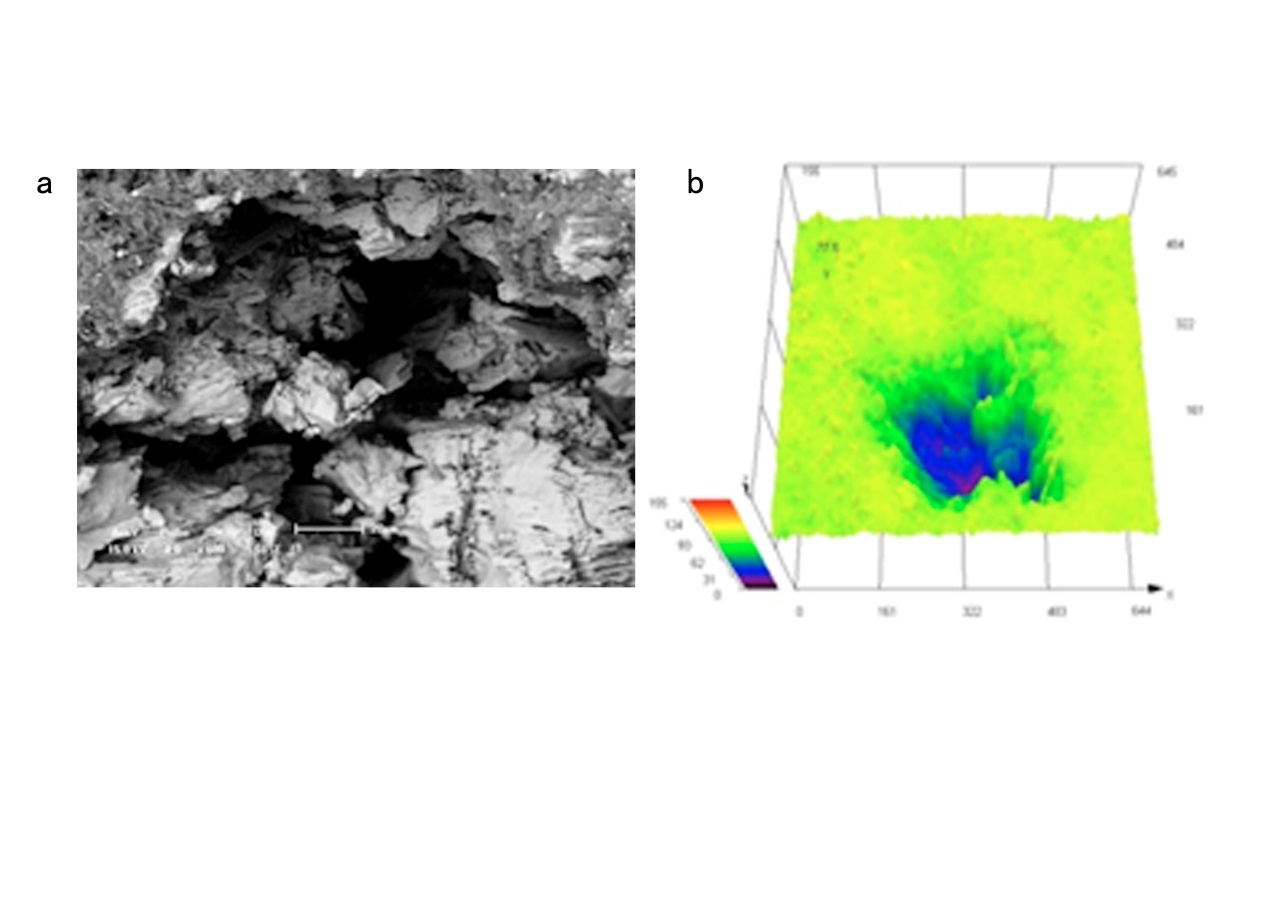
**

**Supplementary Figure S2.** SEM (a) and CLSM (b) images of passive layer controlled samples at the end of bioleaching


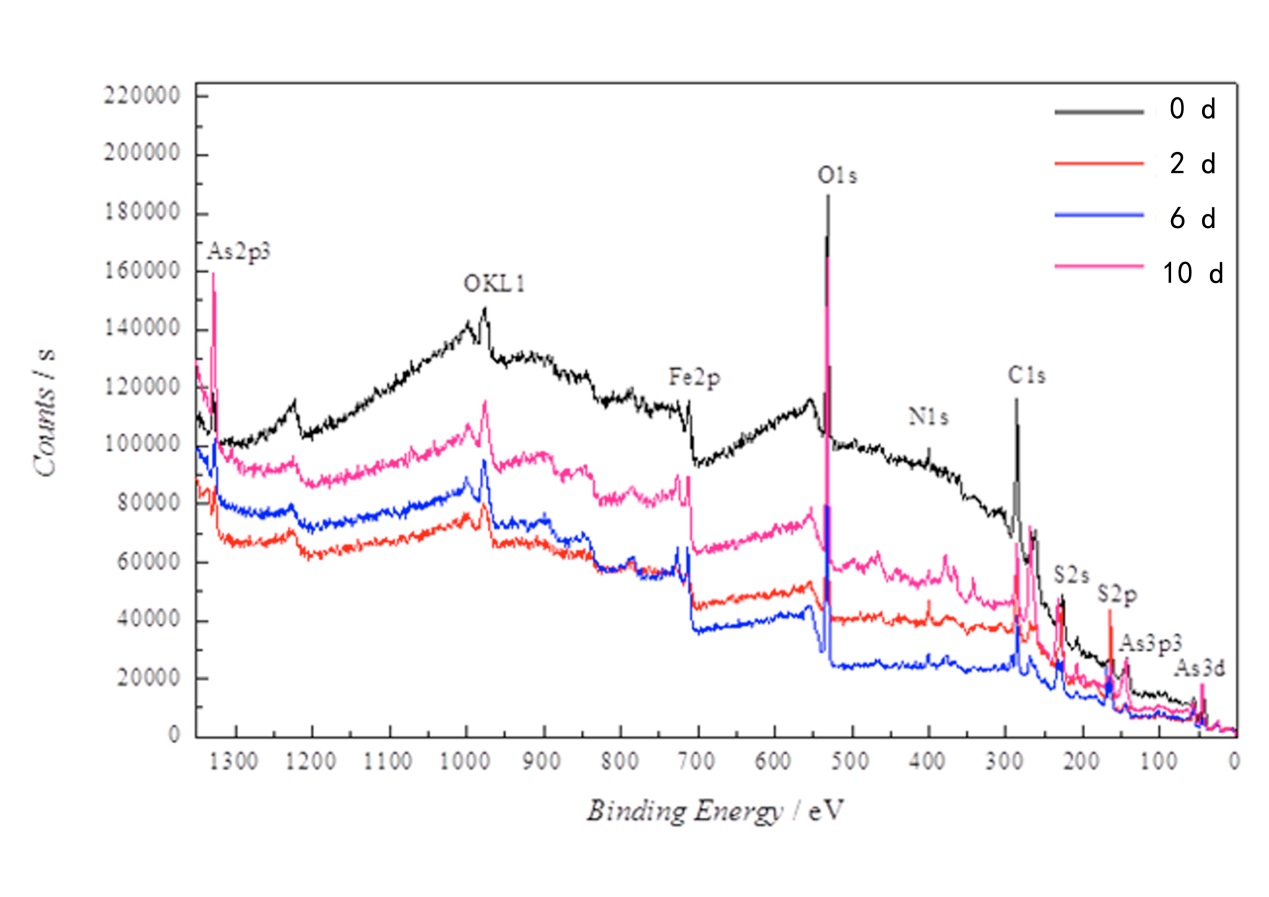


**Supplementary Figure S3.** The survey (full range) XPS spectra of the arsenopyrite samples before bioleaching and bioleached for 2, 6, and 10 d.

## Supplementary Tables

**Supplementary Table S1.** Surface composition (wt, %) of arsenopyrite (0 d) and arsenopyrite after bioleaching for 2 d, 4 d, 6 d, 8 d, and 10 d by EDS. Cells were cultivated at 45 °C and initial pH of 1.5

| Time/d | | 0 | 2 | 4 | 6 | 8 | 10 |
| --- | --- | --- | --- | --- | --- | --- | --- |
| Black | Fe | - | 27.8±1.6 | 14.1±6.7 | 30.4±0.8 | 33.2±3.0 | 25.7±1.7 |
|  | As | - | 24.1±2.8 | 11.5±2.7 | 28.7 ±3.8 | 35.1±0.2 | 52.0±2.5 |
|  | S | - | 48.1±2.1 | 66.3±1.8 | 41.8±11.8 | 31.6±3.3 | 18.0±1.5 |
| Grey | Fe | 25.1±1.7 | 23.4±0.8 | 36.0±7.5 | 45.4±19.1 | 25.8±11.4 | 48.2±3.8 |
|  | As | 54.4±2.0 | 47.9±6.1 | 32.0±8.3 | 40.9±12.7 | 22.8±17.2 | 18.4±8.9 |
|  | S | 16.9±1.6 | 15.3±1.8 | 15.2±3.1 | 10.1±7.6 | 45.1±12.7 | 29.3±5.0 |
| White | Fe | 23.3±3.5 | 36.6±1.7 | 30.3±4.5 | 31.2±16.5 | 26.8±2.1 | 17.9±0.63 |
|  | As | 20.2±3.9 | 32.6±2.2 | 28.2±2.1 | 10.1±4.6 | 13.1±11.5 | 18.8±10.5 |
|  | S | 46.3±4.9 | 24.8±0.6 | 32.1±3.5 | 50.5±22.9 | 58.1±11.4 | 37.13±1.5 |
